# Nicotinamide mononucleotide improves spermatogenesis in aluminium-exposed mice by inhibiting NLRP3-mediated pyroptosis

**DOI:** 10.1371/journal.pone.0339020

**Published:** 2026-01-22

**Authors:** Weihua Nong, Qiumei Huang, Sheng Dou, Yanhong Wei, Yanlun Song, Junli Wang, Xiaocan Lei, Fenglian Yang, Liqiao He

**Affiliations:** 1 Department of Obstetrics and Gynecology, The Affiliated Hospital of Youjiang Medical University for Nationalities, Baise, China; 2 Reproductive Medicine, Guangxi Medical and Health Key Discipline Construction Project of the Affiliated Hospital of Youjiang Medical University for Nationalities, Baise, China; 3 Youjiang Medical University for Nationalities, Baise, China; 4 Department of Histology and Embryology, Clinical Anatomy & Reproductive Medicine Application Institute, Hengyang Medical School, University of South China, Hengyang, China; 5 College of Pharmacy, Youjiang Medical University for Nationalities, Baise, China; University of Hyderabad, INDIA

## Abstract

This study investigates the protective effects of nicotinamide mononucleotide (NMN) against aluminum-induced testicular dysfunction in mice. While previous research has demonstrated the general protective role of NMN in testicular function and highlighted the interaction between aluminum and the NLRP3 inflammasome, the precise mechanisms through which NMN mitigates aluminum-induced reproductive toxicity remain unclear. Our findings show that NMN protects Sertoli cells by inhibiting NLRP3-mediated pyroptosis, which subsequently improves spermatogenesis and testosterone synthesis. We also identify WT1 and GATA4 as key regulators involved in maintaining Sertoli cell integrity and function under aluminum-induced stress. This work provides new insights into the molecular mechanisms of aluminum-induced male infertility and underscores NMN’s potential as a therapeutic strategy for preventing reproductive dysfunction caused by environmental toxicants, such as aluminum. Aluminum exposure disrupts spermatogenesis, yet no effective pharmacological interventions exist to treat aluminum-induced male infertility. In this study, we established an aluminum toxicity model in C57BL/6J mice through intragastric administration of AlCl_3_. We assessed the therapeutic effects of NMN by evaluating testicular histopathology, sperm quality, and serum reproductive hormone levels. Hematoxylin and eosin (H&E) staining revealed significant damage, including reduced seminiferous tubule diameter, disorganized germ cell layers, and a decreased number of germ cells and sperm. Sperm motility was significantly reduced, while the proportion of abnormal sperm increased. However, NMN treatment partially reversed these impairments. Transcriptomic analysis revealed significant upregulation of genes involved in the NLRP3 inflammasome and apoptosis pathways (e.g., NLRP3, GSDMD, caspase-1, and IL-1β) in the AlCl_3_-exposed group. NMN treatment mitigated testicular damage, suppressed NLRP3-mediated pyroptosis in Sertoli cells, and restored serum testosterone levels. Additionally, NMN treatment preserved the expression of key testicular proteins, including WT-1 GATA4, and vimentin. In summary, our study uncovers a novel mechanism by which aluminum exposure impairs spermatogenesis via NLRP3-mediated pyroptosis in testicular cells. We also demonstrate for the first time that NMN can ameliorate aluminum-induced reproductive dysfunction by inhibiting this pathway, offering a potential therapeutic strategy for aluminum-associated male infertility.

## Introduction

Aluminum (Al) is an abundant metal that is considered a potentially hazardous environmental contaminant due to its extensive industrial use and anthropogenic emissions. Its increasing application across diverse industries has been associated with various adverse health outcomes related to occupational and environmental exposure [1]. Aluminum salts are also commonly used as adjuvants in pediatric vaccines, raising ongoing concerns regarding their biological safety [2,3]. Aluminum can enter the human body through dermal absorption, inhalation, ingestion, or intramuscular injection, exerting toxic effects on multiple organ systems, particularly the male reproductive system [4]. The testes are among the primary targets of aluminum toxicity, and even low-level exposure can cause significant reproductive dysfunction, including reduced sperm quality, testicular damage, and hormonal disturbances [4,5,6].

Previous studies have demonstrated that aluminum activates glial cells, sensitizing the DDX3X–NLRP3 pyroptosis pathway and promoting the release of proinflammatory cytokines such as interleukin (IL)-1β and IL-18, which exacerbate neuroinflammation [7]. Animal studies have also shown increased serum levels of TNF-α and IL-6 in aluminum-treated rats, along with histopathological alterations in the testes and epididymides [8]. Similarly, Lokman et al. reported elevated testicular expression of IL-1β and NF-κB in aluminum-exposed rats [9]. These findings suggest that inflammation-related mechanisms, particularly NLRP3-mediated pyroptosis, play a critical role in aluminum-induced male reproductive impairment. However, effective pharmacological strategies to prevent or treat aluminum-induced infertility remain lacking.

Exposure to heavy metals such as lead, cadmium, mercury, and aluminum is widely recognized as a major environmental contributor to male infertility. These metals can accumulate in the testes, epididymis, and accessory glands, inducing oxidative stress, mitochondrial dysfunction, and disruption of the hypothalamic–pituitary–gonadal axis, ultimately impairing spermatogenesis and sperm motility [10]. Testicular injury induced by heavy metals has been linked to increased production of reactive oxygen species (ROS) and inflammatory cytokines, resulting in germ cell apoptosis and reduced sperm function [11]. Moreover, aluminum has been shown to activate the NLRP3 inflammasome, thereby promoting testicular inflammation and pyroptotic cell death [12]. Chronic exposure to heavy metals is therefore a major threat to male reproductive health, as it damages Sertoli and Leydig cells and disrupts spermatogenic homeostasis. Elucidating the mechanisms by which aluminum impairs spermatogenesis and exploring effective therapeutic interventions—such as nicotinamide mononucleotide (NMN)—is thus essential for preventing metal-induced male infertility.

NMN, a key precursor in the biosynthesis of nicotinamide adenine dinucleotide (NAD⁺) [13], is widely used to restore NAD⁺ levels and regulate cellular metabolism. NAD⁺ plays a vital role in controlling inflammation and oxidative stress. In models of intervertebral disc degeneration, NMN has been shown to attenuate IL-1β–induced inflammatory responses by inhibiting the NF-κB pathway in a SIRT1-dependent manner, thereby preventing apoptosis in nucleus pulposus cells [14,15]. Moreover, NMN suppresses the expression of IL-6, IL-1β, and TNF-α by inhibiting NF-κB p65 activation, reducing UV-induced skin inflammation [16]. It also inhibits doxorubicin-induced NLRP3 inflammasome activation, restores antioxidant capacity by increasing glutathione (GSH) and superoxide dismutase (SOD) activity, and reduces oxidative markers such as malondialdehyde (MDA) and ROS [17]. Our previous work demonstrated that NMN restored supporting cell function and cytoskeletal integrity in diabetic mice, significantly improving testicular weight and sperm quality [18]. However, whether NMN can alleviate aluminum-induced reproductive dysfunction remains unclear.

In this study, we established an AlCl_3_-induced mouse model to evaluate the protective effects of NMN against aluminum-related testicular dysfunction. We hypothesize that NMN ameliorates aluminum-induced reproductive impairment by inhibiting NLRP3-mediated pyroptosis, providing a potential therapeutic strategy for aluminum-induced male infertility.

## Methods and materials

### Construction of an aluminum exposure mouse model

A total of 30 mature male C57BL/6J mice, aged 8 weeks and weighing 21−23 g, were purchased from Silaik Jingda Animal Company (License No.: SCXK (Xiang) 2019−0004). The animal experimental protocol was approved by the SCU Laboratory Animal Welfare and Ethics Committee (NO: 2021USA0628). Prior to the experiment, the mice underwent a 1-week acclimatization period. The housing conditions were maintained at a room temperature of 25 ± 2 °C, with a relative humidity of 50% to 70%, and a 12-hour light/dark cycle. During the experimental period, the mice had ad libitum access to food and water. The mice were randomly divided into three groups: the control group, the aluminum exposure group, and the aluminum exposure plus NMN group, with 10 mice in each group. The aluminum exposure group received an intragastric administration of 20 mg/kg AlCl3 (AlCl3·6H2O, sourced from Shanghai Aladdin Biochemical Technology Company, Lot No. A112509). The aluminum exposure plus NMN group received 20 mg/kg AlCl3 followed by an administration of 500 mg/kg NMN (Batch No. XJY01210615, Shenzhen Xigia Biotechnology Co., Shenzhen, China) 30 minutes later. The control group received an equivalent volume of ultrapure water via intragastric administration once daily for a duration of 6 weeks.

### Animal anesthesia, sacrifice, and welfare

All surgical procedures (e.g., for tissue collection) and final sacrifice were performed under anesthesia induced by intraperitoneal injection of urethane (1.0 g/kg). At the end of the experiment, all mice were euthanized by cervical dislocation under deep anesthesia to ensure death without pain. Every effort was made to minimize animal suffering. This included monitoring the mice twice daily for signs of distress (e.g., reduced mobility, abnormal posture, or significant weight loss), providing soft bedding, and ensuring ad libitum access to food and water in a controlled environment throughout the study [18–21].

### Sperm analysis

After six weeks of treatment, the mice were anesthetized, and relevant tissues were collected to calculate various parameters. One side of the epididymis was placed in 1.5 mL of 37°C physiological saline and thoroughly cut open. The sample was incubated at 37°C for 15 minutes to allow the sperm to swim out completely. The sperm suspension was then transferred to a counting chamber for analysis. Sperm morphology was examined using a rapid staining kit (Solarbio, China), and sperm counts and the number of abnormal sperm were quantitatively analyzed under a microscope using the counting chamber. A total of 200 sperm were counted, and the percentage of abnormal sperm was calculated. Specific observational methods were conducted according to the previously described procedures [22].

### Histological analysis of testicular and epididymal tissues

Testicular and epididymal tissues were fixed in 4% formaldehyde for 24 hours, followed by paraffin embedding and sectioning into 5 µm thick slices for histological analysis. Hematoxylin and eosin (H&E) staining was performed to examine the morphology of the seminiferous tubules and epididymis in the testicular tissue sections. Subsequently, 50 seminiferous tubules were randomly selected from each group under a microscope, and their areas and diameters were measured and statistically analyzed.

### Serum detection of reproductive hormones in mice

After anesthetizing the mice, blood was collected by excising the eyeballs. The blood was allowed to stand at room temperature (23 ± 2 °C) for 30 minutes and then centrifuged at 4 °C at 3000 rpm for 15 minutes. The serum supernatant was separated by the Northern Beijing Institute of Biotechnology for radioimmunoassay to measure testosterone levels. An enzyme-linked immunosorbent assay (ELISA) kit was used to determine testosterone levels in the cell culture supernatant according to the manufacturer’s instructions. The protein content was measured at 450 nm.

### Transcriptome sequencing and bioinformatic analysis of mouse testes

Testicular RNA sequencing was conducted by Novogene (Beijing, China). In brief, to assess the purity and concentration of RNA, a NanoPhotometer® spectrophotometer (Implen Inc., CA, USA) and a Qubit® RNA Assay Kit in a Qubit® 2.0 Fluorometer (Life Technologies, CA, USA) were utilized. Following the manufacturer’s instructions, an RNA sequencing library was created using the NEBNext® Ultra™ RNA Library Prep Kit for Illumina® (California, USA), with each sample containing 3 µg of RNA. Index codes were incorporated into the attribute sequences of each sample. The indexed samples were clustered using the TruSeq PE Cluster Kit v3 cBot-HS (Illumina, California, USA) on the cBot clustering generation system. The prepared libraries were sequenced on the Illumina HiSeq platform, generating paired-end reads of 150 bp. Differential expression analysis between groups was performed using the DESeq2 R package (1.10.1). DESeq2 adjusted the p-values of the genes to determine which genes exhibited differential expression. Statistical enrichment of differentially expressed genes (DEGs) in KEGG pathways was examined using the Cluster Profiler R package (http://www.genome.jp/kegg/).

### Quantitative real-time PCR (qRT-PCR)

Real-time quantitative reverse transcription PCR (qRT-PCR) was performed to detect mRNA expression levels. Total RNA was extracted from mouse testicular tissue using TRIzol reagent (Invitrogen, Carlsbad, CA, USA). Subsequently, RNA was reverse transcribed into complementary DNA (cDNA) using the TransGen One-Step gDNA Removal and cDNA Synthesis Super Mix (TransGen, Beijing, China). GAPDH was used as a reference for mRNA expression. Real-time quantitative PCR was conducted on an ABI7900 PCR system (Applied Biosystems, Foster City, CA, USA) using SYBR Green qPCR Master Mix (Thermo Fisher Scientific, Cat. No. 4309155). The primer sequences are listed in Table 1.

**Table 1:** Primers sequences used as target and reference genes used in qPCR reactions.

Gene	Primer sequence (5’-3’)	Accession no.
Wt1	F: ATCCGCAACCAAGGATACAG R: GGTCCTCGTGTTTGAAGGAA	NM_144783.4
GATA4	F: ATGCCTGTGGCCTCTATCAC R: GGTGGTGGTAGTCTGGCAGT	NM_008092.2
Vimentin	F: GCTGCGAGAGAAATTGCAGGA R: CCACTTTCCGTTCAAGGTCAAG	NM_011701.4
NLRP3	F: ATCAACAGGCGAGACCTCTG R: GTCCTCCTGGCATACCATAGA	NM_145827.2
Caspace-1	F: TCATCTATGATGGCAAGGAGTG R: CAAAGTCAATCATGCGGACATC	NM_009807.3
GSDMD	F:CGATGGGAACATTCAGGGCAGAG R:ACACATTCATGGAGGCACTGGAAC	NM_026960.4
IL1-β	F: ACCTTCCAGGATGAGGACATGA R: GATTCTTTCCTTTGAGGCCCA	NM_008361.1
TNF-α	F:ACTGAACTTCGGGGTGATCG R: CCACTTGGTGGTTTGTGAGTG	NM_001278601.1
IL-6	F:CTGGTCTTCTGGAGTACCATAGC R:GTGACTCCAGCTTATCTCTTGGT	NM_001314054.1
Occludin	F:TTTCAGGTGAATGGGTCACCG R: GAGCAAAATGTCCAGGCTCC	NM_001360536.1
ZO-1	F:TTCCCGGACTTTTGTCCCAC R:CTGGCGGACATCTTGTCTCT	NM_001163574

### Western blot analysis

The testicular lysate was prepared by combining 300 μL of RIPA lysis buffer (CWBIO, Beijing, China) with 6 μL of PMSF (Solarbio) and three steel balls with a diameter of 3.2 mm in a 1.5 mL EP tube. The tissue was homogenized using a tissue lyser (Tissuelyser-24). The protein concentration was determined using a BCA protein assay kit (CWBIO), following denaturation of the proteins by heating at 100°C for 10 minutes. Denatured proteins were separated by 10% SDS-PAGE and subsequently transferred to a polyvinylidene fluoride membrane. The membrane was blocked for 2 hours with PBST (phosphate-buffered saline containing 0.1% Tween-20) supplemented with 5% non-fat dry milk. After washing with PBST, the membrane was incubated overnight at 4°C with primary antibodies against Tubulin, Vimentin, WT-1 GATA4, NLRP3, GSDMD, caspase-1, and IL-1β. Subsequently, the membrane was incubated for 2 hours at room temperature with HRP-conjugated affinity-purified goat anti-mouse IgG (H + L) or goat anti-rabbit IgG (H + L). Finally, ECL (CW0049M, CWBIO) was added, and the protein bands were detected using the T-5500 chemiluminescence imaging system. The antibodies used in the experiment are listed in Table 2.

**Table 2:** Antibody used as target and reference used in immunohistochemistry and Western Blot reactions.

Antibody	Accession no.	Company
HRP-conjugated affinipure goat anti-mouse IgG (H + L)	SA00001–1	Proteintech Co.,Ltd.
HRP-conjugated affinipure goat anti-rabbit IgG (H + L)	SA00001–2	Proteintech Co.,Ltd.
Wt1 Polyclonal antibody	12609-1-AP	Proteintech Co.,Ltd.
GATA4-Specific Polyclonal antibody	19530-1-AP	Proteintech Co.,Ltd.
Vimentin Polyclonal antibody	10366-1-AP	Proteintech Co.,Ltd.
NLRP3 Monoclonal antibody	68102-1-Ig	Proteintech Co.,Ltd.
GSDMD Polyclonal antibody	20770-1-AP	Proteintech Co.,Ltd.
Caspase1/p20/p10 Polyclonal antibody	22915-1-AP	Proteintech Co.,Ltd.
IL-1 beta Polyclonal antibody	26048-1-AP	Proteintech Co.,Ltd.
Alpha Tubulin Monoclonal antibody	66031-1-Ig	Proteintech Co.,Ltd.

### Immunohistochemical analysis

Paraffin sections were hydrated using an ethanol gradient and subjected to high-pressure incubation for 3 minutes in a pH 6.0 citrate buffer to retrieve antigens. Endogenous peroxidase blocking agent was applied, followed by washing with PBS. A nonspecific staining blocking agent was then added, along with the corresponding primary antibody solution. The samples were covered with a wet box lid and incubated overnight at 4°C. The primary antibody solution was washed out with PBS, and a secondary antibody was introduced and incubated for 20 minutes. Following another PBS wash to remove excess secondary antibody, DAB chromogen was applied for staining. After thorough washing with water to remove residual DAB, the samples were stained with hematoxylin, washed, blued, dehydrated, and mounted. Each group was analyzed and counted for the number of positive cells in 50 seminiferous tubules under a microscope. Antibodies used are listed in Table 2.

### Statistical analysis

Data were analyzed using GraphPad Prism 8.0 (GraphPad Software, La Jolla, USA). Continuous variables are expressed as mean ± standard deviation (SD), and one-way analysis of variance (ANOVA) was employed. For pairwise comparisons between groups, the Student-Newman-Keuls (SNK) test was used when variances were homogeneous, while the Games-Howell test was applied when variances were heterogeneous. A p-value of ≤ 0.05 was considered statistically significant.

## Results

### Effects of NMN treatment on testicular and epididymal morphology and sperm quality in aluminum-exposed mice

An aluminum exposure model was established via oral AlCl_3_ administration to investigate NMN’s effects on body weight, testicular weight, and semen quality in aluminum-exposed mice. After one week of acclimation feeding, male mice were randomly assigned to groups. The AlCl_3_ intervention group exhibited reduced body weight, which gradually increased after NMN supplementation, aligning with the control group. Testicular weighing revealed reduced weights in the AlCl_3_-treated group (0.16 ± 0.01 g, p < 0.01) compared to the control group. NMN supplementation increased testicular weight (0.19 ± 0.01 g, p < 0.01). Sperm quality assessment in the AlCl_3_-treated group revealed decreased motility, reduced sperm count, and increased abnormal sperm proportion, with more pronounced abnormalities than the control group. All parameters improved after NMN supplementation. NMN treatment significantly increased sperm count in aluminum-exposed mice, improved body weight, testicular weight, and sperm motility, while reducing both the proportion and severity of abnormal sperm (Table 3).

**Table 3:** Effects of NMN treatment on body weight, testis weight and semen quality of the mice.

Group	body weight(g)		Testis weight(g)	Sperm count (×10^6^)	Sperm fastforward movement rate(%)	Sperm malformation rate(%)
	Pre-experimental	Post-experimental				
Ctrl	22.00 ± 0.58	27.35 ± 0.67	0.21 ± 0.01	23.61 ± 2.24	35.53 ± 1.99	30.70 ± 0.75
AlCl_3_	21.70 ± 0.43	23.73 ± 0.27^a^	0.16 ± 0.01^a^	10.06 ± 1.10^a^	7.90 ± 1.13^a^	45.41 ± 2.24^a^
AlCl_3_ + NMN	21.95 ± 0.33	25.22 ± 0.85^b^	0.19 ± 0.01^b^	13.89 ± 0.93^b^	23.15 ± 1.50^b^	35.24 ± 2.12^b^

Compared with control group, a: P < 0.01; Compared with aluminum exposure group b: P < 0.01.

### Effects of NMN on reproductive hormones in aluminum-exposed mice

**Fig 1:**
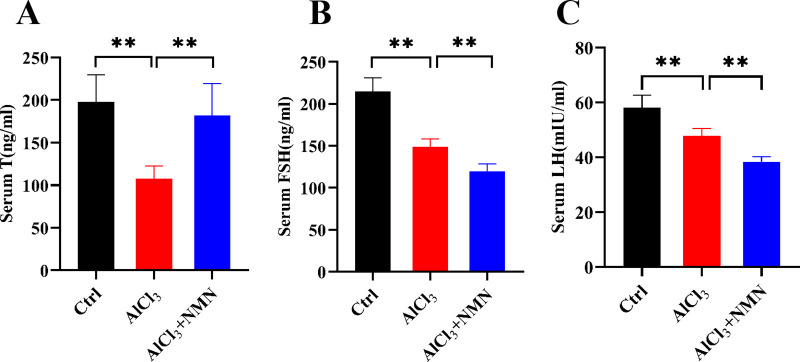
Effects of NMN intervention on testosterone, FSH and LH in mice exposed to aluminum. A) Serum testosterone; B) Serum testosterone FSH; C) Serum LH. Testosterone (T), Follicle-stimulating hormone(FSH), luteinizing hormone (LH); **p < 0.01.

To evaluate the impact of aluminum exposure on reproductive health in mice, we measured reproductive hormones. Results showed that serum testosterone levels decreased in the AlCl_3_ intervention group compared to controls, but increased after NMN supplementation (Fig 1A). Similar to testosterone, serum FSH and LH concentrations were significantly reduced in the AlCl_3_-treated group. Following NMN treatment, female hormones FSH and LH concentrations remained significantly decreased (Fig 1B, C), potentially related to feedback regulation of the HPG axis.

### Effects of NMN treatment on the morphology of testes and epididymis in mice exposed to aluminum

**Fig 2:**
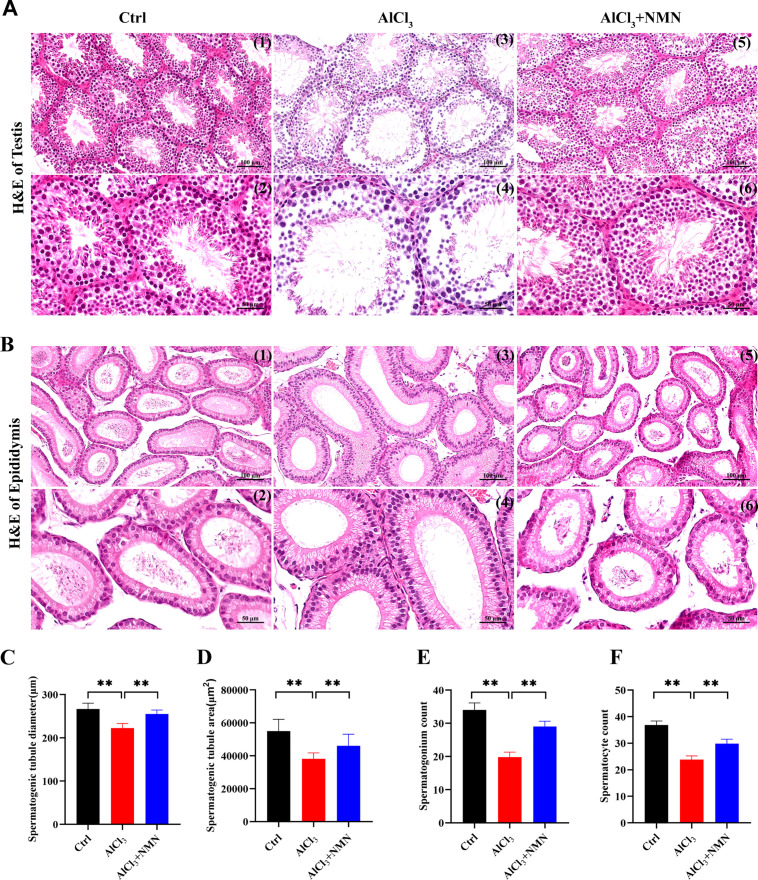
Effect of NMN intervention on testis and epididymis of mice exposed to aluminum. A) HE staining of mouse testis; B) HE staining of mouse epididymis; C) Diameter of mouse spermatogenic tubule; D)Mouse spermatogenic tubule area; E-F) Spermatogonium and spermatocyte count. **p < 0.01.

The morphology of the testes and epididymis was assessed across different groups of mice. In the control group, the testes exhibited normal structural integrity, with intact seminiferous tubules and orderly arrangement of germ cells at various stages of division. A significant number of differentiated and mature sperm were visible within the lumen (Fig 2A, B). In contrast, aluminum-exposed mice showed atrophy of the seminiferous tubules, disorganized structures, and a reduced number of germ cells with sparse arrangement (Fig 2A). Notably, very few sperm were observed in the epididymis of aluminum-exposed mice (Fig 2B, E and F). However, following NMN treatment, these pathological changes showed varying degrees of improvement (Fig 2A–F).

### Impact of NMN intervention on the function of testicular sertoli cells in aluminum-exposed mice

**Fig 3:**
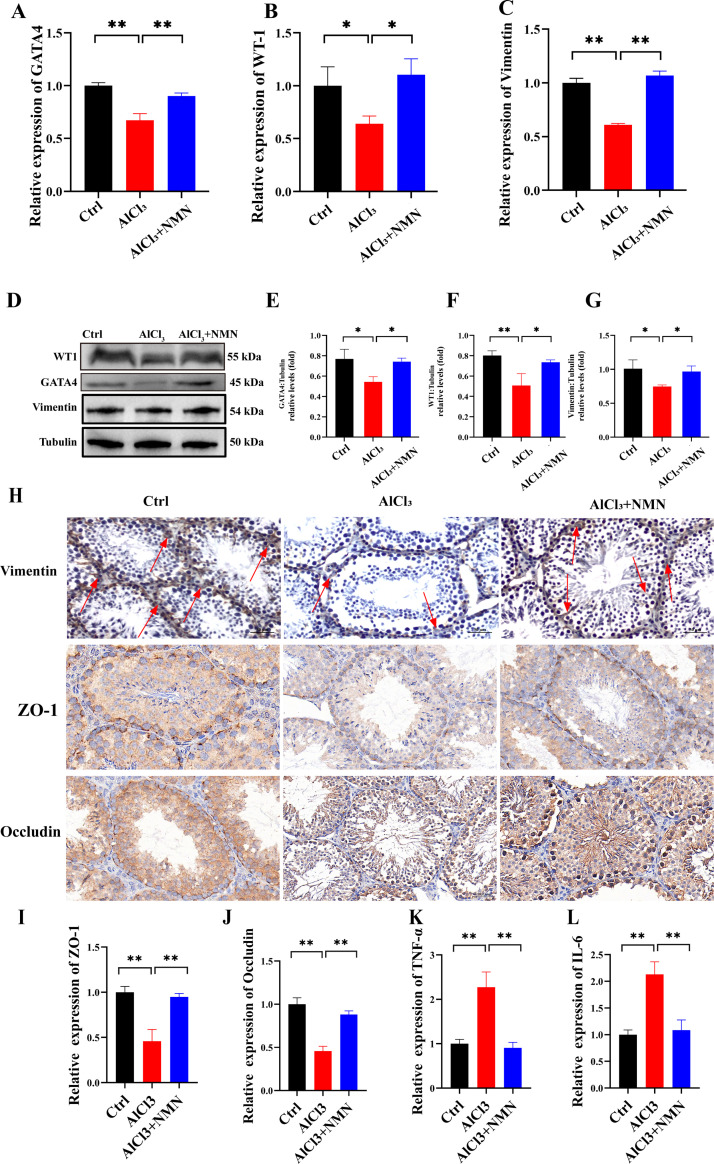
Effect of NMN intervention on function of supporting cells in mice exposed to aluminum. A–C) qPCR validation of NMN intervention effects on GATA4, WT-1 and Vimentin mRNA expression in aluminum-exposed mouse testes; D–G) Western Blotting assessment of NMN intervention effects on testicular supporting cell function in aluminum-exposed mice; H) Immunohistochemical detection of Vimentin, ZO-1, and Occludin expression in mouse testicular tissue; I–L) qPCR validation of NMN intervention effects on ZO-1, Occludin, TNF-α, and IL-6 in aluminum-exposed mouse testes. *p < 0.05, **p < 0.01.

Sertoli Cells play a crucial role in spermatogenesis. To investigate whether the function and structure of testicular Sertoli Cells in aluminum-exposed mice are compromised, we employed qRT-PCR, Western blotting, and immunohistochemistry to assess the changes in mRNA and protein expression of WT-1 GATA4, and Vimentin. Compared to the control group, the mRNA expression levels of SCs marker genes (GATA4, WT-1 Vimentin) were significantly reduced in the testes of aluminum-exposed mice. However, NMN treatment significantly increased the expression of GATA4, WT-1 and Vimentin mRNA in the testes compared to aluminum-exposed mice (Fig 3A–C). The results of Western blot analysis of SCs marker gene expression corroborated our qRT-PCR findings (Fig 3D–G). Immunohistochemical analysis of testes from different treatment groups revealed a decrease in the number of SCs in the testes of aluminum-exposed mice compared to the control group (Fig 3H). Red arrows indicate cells with positive signals. The number of vimentin-positive cells was significantly higher in the AlCl_3_ + NMN group compared to the AlCl_3_ group (Fig 3H).

To assess the effects of aluminum exposure and NMN treatment on blood-testis barrier (BTB) integrity, immunohistochemical staining was used to detect the expression of tight junction proteins occludin and ZO-1 in mouse testes. As shown in Fig 3H, both occludin and ZO-1 expression were significantly reduced in the AlCl_3_ group, indicating impaired BTB structure. qPCR results were consistent with this finding (Fig 3I, J). Conversely, NMN administration restored their expression levels to near-control group levels. These findings indicate that aluminum exposure compromises blood-testis barrier integrity, while NMN supplementation protects and stabilizes tight junction dynamics within Sertoli cells.

### Supplementation with NMN can alter the transcriptional profile in mice exposed to aluminum

**Fig 4:**
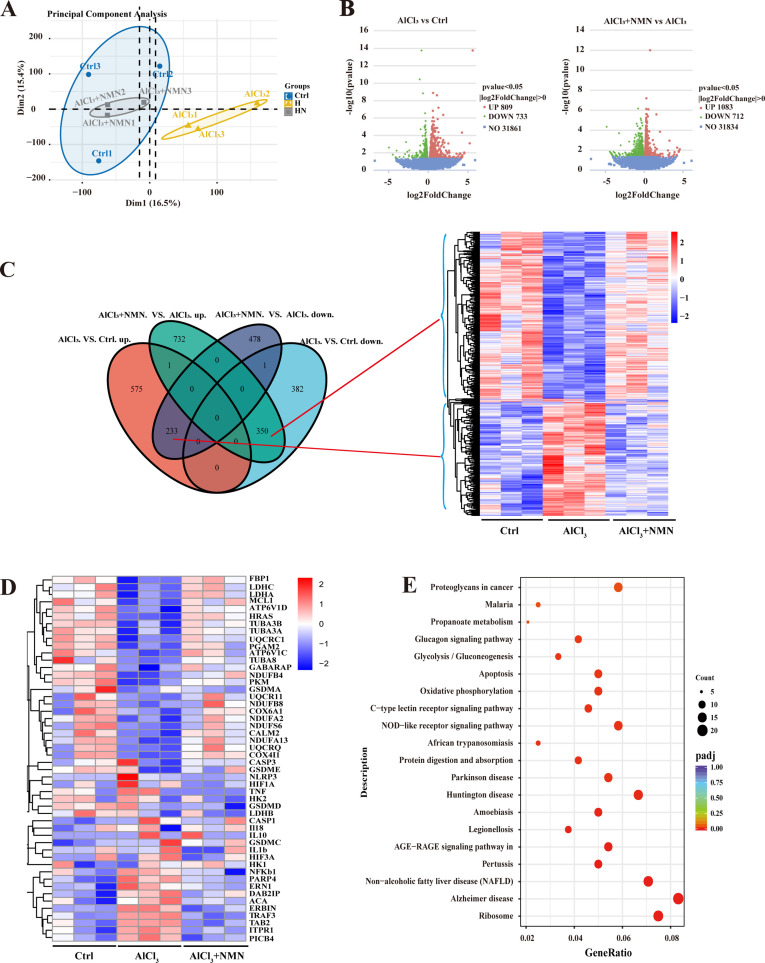
NMN Modulates Testis Gene Expression in Aluminum-Exposed Mice. Testicular tissues from the control, high aluminum dose, and NMN intervention groups were analyzed through RNA sequencing. A) PCA analysis of testis gene expression is shown, with each point representing a sample. B) Volcano map comparison of gene expression between pairs (n = 3). C) Venn diagrams illustrate the differentially expressed genes between groups, and heat maps of the differentially expressed genes in clusters. D) Cluster analysis of genes related to the NOD-like receptor signaling pathway. E) KEGG pathway enrichment analysis of differentially expressed genes across the three groups.

To further investigate the molecular mechanisms by which NMN improves spermatogenesis in aluminum-exposed mice, we conducted RNA sequencing (RNA-seq) analysis on testicular tissue. Principal component analysis (PCA) of all expressed genes revealed significant changes in the transcriptome of the testicular tissue from mice treated with AlCl_3_. Notably, NMN treatment partially ameliorated these changes (Fig 4A). Visualization through a volcano plot (Fig 4B) showed that compared to the Ctrl, the AlCl_3_ treatment group had 809 upregulated genes and 733 downregulated genes. Following NMN supplementation, the AlCl_3_ treatment group exhibited 1,083 upregulated genes and 712 downregulated genes (Fig 4B). Venn diagram analysis and clustered heatmap analysis (Fig 3C) were employed to integrate data across groups. Notably, the AlCl_3_ treatment group displayed 233 differentially expressed genes (DEGs) that were upregulated compared to the control group, while these genes were downregulated in the AlCl_3_ + NMN group. Conversely, 350 DEGs that were downregulated in the AlCl_3_ treatment group were upregulated in the AlCl_3_ + NMN group. Our findings suggest that these genes may play a crucial regulatory role in NMN’s ability to alleviate spermatogenic impairment caused by aluminum exposure. Further clustering analysis through heatmap visualization revealed that genes such as NLRP3, GSDMD, caspase-1, and IL-1β were upregulated in the AlCl_3_ treatment group compared to the control group but were improved following NMN treatment (Fig 4D). To deepen our understanding of the biological logic behind these changes in gene expression, we conducted KEGG enrichment analysis, which indicated that these DEGs were primarily enriched in signaling pathways related to NOD-like receptors, apoptosis, and oxidative stress (Fig 4E). This molecular mechanism elucidates how NMN improves reproductive dysfunction in aluminum-exposed mice by inhibiting NLRP3-mediated pyroptosis in germ cells.

### NMN intervention alleviates inflammatory responses and pyroptosis levels in testicular tissues of aluminum-exposed mice

**Fig 5:**
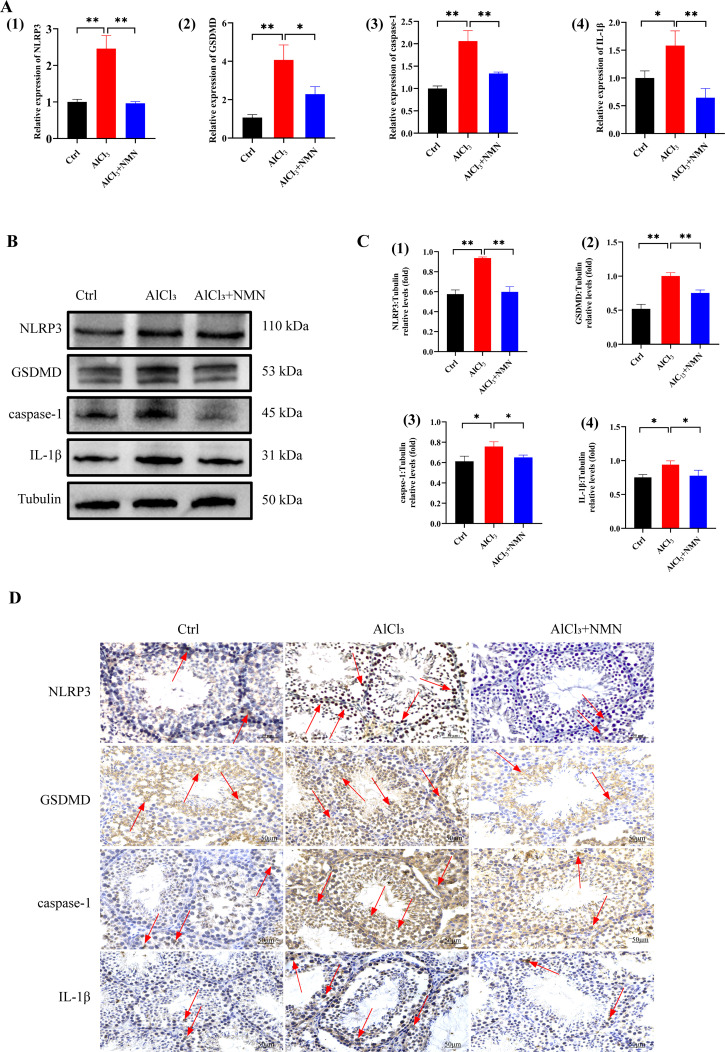
Effect of NMN intervention on NLRP3 and inflammation related genes in testis of mice exposed to aluminum. A) Effect of NMN intervention on the expression of NLRP3,GSDMD, caspase-1 and IL-1β mRAN in the testis of aluminium-exposed mice; B) Western Blotting detected the effects of NMN intervention on the expression of NLRP3 and inflammation-related gene proteins in the testis of mice exposed to aluminum; C)Image J Analysis of the effects of NMN intervention on the expression of NLRP3,GSDMD, caspase-1 and IL-1β proteins in the testis of aluminium-exposed mice; D) Immunohistochemical detection of NMN intervention on the expression of NLRP3 and inflammation-related gene proteins in testis of aluminum-exposed mice.

To further evaluate inflammatory responses, we detected TNF-α and IL-6 expression levels in testicular tissues via qRT-PCR. As shown in Fig 3K, L, compared with the control group, mRNA expression levels of both TNF-α and IL-6 were significantly elevated in the AlCl_3_ group. Notably, NMN treatment significantly reduced the expression levels of these cytokines, restoring them to levels comparable to the control group. Transcriptomic analysis of testicular tissue revealed that AlCl_3_ treatment significantly upregulated the expression of inflammation-related and pyroptosis-associated factors. Key regulatory molecules and effectors of testicular pyroptosis (including NLRP3, GSDMD, caspase-1, and IL-1β) showed an upward trend in the AlCl_3_-treated group, but their expression levels were significantly reduced after NMN intervention. Expression levels of NLRP3, caspase-1, GSDMD, and IL-1β in testicular tissues across groups were detected via qRT-PCR, Western blotting, and IHC techniques, revealing expression trends consistent with transcriptomic analysis. As shown in Fig 5A, qRT-PCR results indicated significantly elevated mRNA expression levels of key regulatory molecules and effector factors (NLRP3, caspase-1, GSDMD, and IL-1β) in the testes of AlCl_3_-treated mice, which exhibited a downregulated trend after NMN intervention. Western blot results corroborated the qRT-PCR analysis trend (Fig 4B, C). Furthermore, immunohistochemical results confirmed the presence of pyroptosis-related factor expression in testicular tissue, which was significantly elevated in the AlCl_3_-treated group compared to the control group and decreased following NMN intervention (Fig 5D).

### NMN intervention significantly reverses AlCl_3_-induced damage to mouse spermatogenic cells

**Fig 6:**
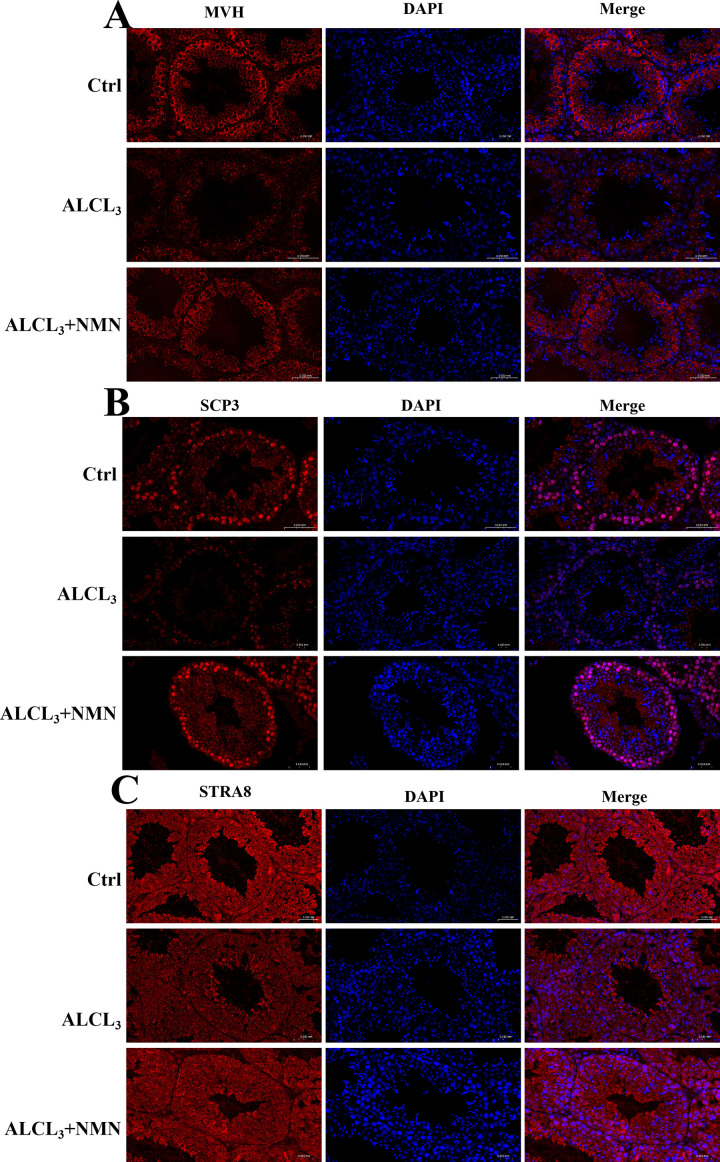
Effect of NMN Intervention on Marker Expression at Different Developmental Stages of Spermatogenic Cells in AlCl_3_-Exposed Mice. Immunofluorescence staining was used to detect the expression of germ cell markers at different developmental stages. A) Shows that the pan-germ cell marker MVH was significantly downregulated in the AlCl_3_-exposed group, with expression restored in the AlCl_3_ + NMN group; B) The pro-meiotic marker STRA8 showed no significant differences among the control, AlCl_3_, and AlCl_3_ + NMN groups; C) Spermatocyte meiosis marker SCP3 showed significantly reduced expression in the AlCl_3_ group, with expression restored in the AlCl_3_ + NMN group following NMN intervention. Results indicate aluminum exposure primarily impacts the meiosis stage of germ cells, while NMN maintains spermatocyte meiosis integrity to exert protective effects.

To precisely identify the developmental stage at which germ cell loss occurs, this study employed immunofluorescence staining to systematically detect germ cell-specific markers at different developmental stages. Results showed that the pan-germ cell marker MVH exhibited significantly reduced expression in the AlCl_3_-exposed group, while recovery to near-normal levels was observed in the AlCl_3_ + NMN group (Fig 6A). Notably, the pro-meiotic marker STRA8 showed no significant differences among the three groups (control, AlCl_3_, and AlCl_3_ + NMN), indicating that spermatocyte differentiation remained relatively intact under these conditions (Fig 6B). Conversely, the meiosis-specific marker SCP3 in spermatocytes was significantly weakened in the AlCl_3_ group, while NMN intervention effectively restored its expression levels (Fig 6C). These findings suggest that aluminum exposure primarily impacts the meiotic stage of germ cells, while NMN exerts significant cytoprotective effects by maintaining meiotic integrity in spermatocytes.

## Discussion

Aluminum is a prevalent environmental toxicant that can enter the body via inhalation, ingestion, dermal contact, and intramuscular injection. Accumulation of aluminum can exert toxic effects on various organ systems, with the male reproductive system being one of its primary targets. Exposure to aluminum can lead to impaired fertility and significantly reduced sperm quality [4]. In this study, mice exposed to AlCl_3_ exhibited severe histological damage, including reduced seminiferous tubule area, morphological disruption, and a decrease in spermatogonia, primary spermatocytes, Sertoli cells, and sperm. Additionally, there was a significant reduction in testicular and epididymal weights, as well as decreased sperm motility and an increase in abnormal sperm. These findings align with previous reports indicating that aluminum exposure impairs spermatogenesis and epididymal sperm maturation. Notably, NMN treatment effectively ameliorated these reproductive damages, suggesting that its protective effects extend beyond cellular and molecular levels to functional sperm outcomes. The improvement in sperm quality likely results from the restoration of Sertoli cell integrity, reduced oxidative stress, and normalization of testosterone levels, all contributing to enhanced spermatogenic efficiency.

Our study investigates the protective role of nicotinamide mononucleotide (NMN) against aluminum-induced testicular dysfunction, focusing on its interaction with the NLRP3 inflammasome. While previous studies have indicated NMN’s protective effects on testicular function [23,24] and the role of NLRP3 in aluminum toxicity [25,26], our research uncovers a novel mechanism by which NMN alleviates aluminum-induced reproductive dysfunction. Specifically, we show that NMN mitigates testicular damage by inhibiting NLRP3-mediated pyroptosis in Sertoli cells, a pathway not fully explored in the context of aluminum exposure.

This study identifies NMN as an effective inhibitor of NLRP3, GSDMD, caspase-1, and IL-1β expression, key components of the inflammasome pathway. By reducing the activation of these inflammatory mediators, NMN restores spermatogenesis, improves testosterone synthesis, and enhances overall testicular function. Furthermore, we highlight the roles of WT1 and GATA4 in maintaining Sertoli cell integrity and their restoration following NMN treatment, contributing to an improved testicular microenvironment and fertility outcomes.

Thus, our findings represent a significant advancement in understanding the molecular mechanisms underlying aluminum-induced reproductive toxicity and provide evidence supporting NMN as a potential therapeutic agent for male infertility caused by environmental toxicants like aluminum.

Sertoli cells (SCs), located within the seminiferous tubules, are crucial for the development and maturation of germ cells [27]. The integrity of the blood-testis barrier (BTB) is essential for spermatogenesis, as it provides a specialized microenvironment for germ cell development and protects against toxic insults. Occludin and ZO-1 are critical components of the Sertoli cell tight junction complex that regulate BTB permeability. Our results show a marked reduction in these junctional proteins following aluminum exposure, suggesting BTB disruption and impaired Sertoli cell junctional communication. NMN treatment effectively restored Occludin and ZO-1 expression, indicating its role in maintaining BTB stability. These findings align with previous evidence that NAD ⁺ -dependent signaling enhances tight junction assembly and antioxidative capacity in epithelial barriers. Therefore, NMN’s protective effects on BTB integrity may represent a key mechanism by which it preserves spermatogenesis under aluminum toxicity.

Previous research has identified aluminum accumulation in Sertoli cells of exposed mice, with transmission electron microscopy revealing disrupted tight junctions between Sertoli cells [28,29]. Vimentin, a type III intermediate filament, is a major structural protein in the Sertoli cell cytoskeleton. During normal spermatogenesis, Vimentin is the predominant protein in the SC cytoskeleton, and its distribution pattern is directly related to the morphological integrity of the germinal epithelium [30]. The downregulation of Vimentin is associated with decreased spermatogenesis [31]. GATA4 and WT1 are key transcription factors for Sertoli cell differentiation and function. Their downregulation in aluminum-exposed mice indicates impaired Sertoli cell support, which NMN treatment alleviates by restoring their expression [32–37].

Our findings indicate that mice exposed to AlCl_3_ exhibited a significant downregulation of Vimentin expression, which may disrupt the connections between Sertoli cells and germ cells, leading to significant sloughing of the germinal epithelium [34] and a reduction in spermatogonia, primary spermatocytes, and supporting cells. GATA4 is considered a specific marker for supporting cells during development and adulthood [32,33]. Numerous studies have shown that GATA4 works synergistically with the WT1 gene to promote Sertoli cell function, male sexual differentiation, and testosterone production [36–39]. Our results demonstrate that mRNA and protein expressions of WT1 and GATA4 were significantly reduced in the testicular tissue of mice exposed to aluminum. NMN upregulated the expression levels of WT1 and GATA4 in AlCl_3_-treated mice, mitigating the destruction of tubule integrity and Sertoli cell connections, ultimately improving the testicular dysfunction induced by AlCl_3_.

Further immunofluorescence analysis revealed that germ cell loss was most pronounced during the meiotic stage, rather than the pre-meiotic phase. MVH expression was markedly downregulated following aluminum exposure, indicating overall germ cell depletion, while STRA8 expression remained relatively stable across groups, suggesting preserved spermatogonial differentiation. However, the marked reduction in SCP3, a synaptonemal complex protein essential for meiosis, confirmed that aluminum primarily disrupts spermatocyte meiosis. NMN supplementation restored SCP3 expression, implying that NMN preserves meiotic progression by protecting Sertoli cell function and reducing inflammatory and oxidative stress in the testicular microenvironment.

To further investigate the mechanism by which NMN ameliorates reproductive damage due to aluminum exposure, we performed transcriptome sequencing (RNA-seq) analysis on testicular tissue. While NMN’s protective effects on testes in diabetic/aged models and aluminum-NLRP3 links in neural systems have been reported, our work uncovers a previously unrecognized role of NLRP3-GSDMD pyroptosis in aluminum-induced spermatogenesis impairment. Crucially, we identify NMN as an effective inhibitor of this pathway specifically in testicular tissue and establish WT1/GATA4 as key regulators in maintaining the spermatogenic microenvironment upon aluminum exposure (Fig 3). These findings expand the mechanistic understanding of reproductive toxicity and highlight NMN’s translational potential.

The results revealed significant upregulation of NLRP3, GSDMD, caspase-1, and IL-1β in the AlCl_3_ treatment group compared to the control group, which improved after NMN supplementation. Studies indicate that non-infectious testicular inflammation can also lead to decreased Sertoli cell function [40]. Additionally, high expression of NLRP3 in rat Sertoli cells has been shown to activate caspase-1, promoting the secretion of inflammatory cytokines IL-1β and IL-18 [41]. There is substantial evidence that pyroptosis plays a critical role in male reproductive disorders, with extensive research confirming that the NLRP3 inflammasome is a key regulatory factor in pyroptosis [42]. The NLRP3 inflammasome is a multiprotein complex composed of the intracellular innate immune receptor NLRP3, apoptosis-associated speck-like protein (ASC), and the protease caspase-1. Activation of the NLRP3 inflammasome induces the maturation and secretion of IL-1β and IL-18, subsequently triggering pyroptosis [43,44]. Consistently, our findings indicate that AlCl_3_-induced inflammation activates NLRP3-mediated pyroptosis in Sertoli cells.

Although caspase-3 and caspase-9 are pivotal in apoptotic cell death, our study focused on caspase-1 due to its specific role in NLRP3 inflammasome-mediated pyroptosis, a process characterized by inflammatory cytokine release and membrane rupture, which aligns with the histopathological observations in aluminum-exposed testes (Fig 2). Notably, caspase-1 activation directly cleaves GSDMD to execute pyroptosis, whereas caspase-3/-9 operate in distinct apoptotic pathways. This mechanistic specificity justifies our emphasis on caspase-1 as the key effector in aluminum-induced testicular injury.

Transcriptome sequencing results showed upregulation of NLRP3, GSDMD, caspase-1, and IL-1β in the AlCl_3_ treatment group, which were significantly downregulated following NMN supplementation. These results suggest that AlCl_3_-induced impairment of spermatogenic function in male mice is directly related to testicular inflammatory responses and activation of pyroptosis pathways, with NLRP3, GSDMD, caspase-1, and IL-1β potentially serving as key targets for treating AlCl_3_-induced testicular dysfunction. Molecular validation through Western blot and quantitative PCR confirmed these findings, showing that NMN treatment significantly reduced the expression levels of these inflammatory mediators. Immunohistochemical results were consistent with the transcriptome sequencing, Western blot, and PCR findings, further demonstrating that NMN improves male reproductive dysfunction caused by aluminum exposure by suppressing NLRP3, GSDMD, caspase-1, and IL-1β expression.

In conclusion, this study established a mouse model.

## Conclusion

NMN mitigates NLRP3 inflammasome activation-induced pyroptosis, reduces inflammatory factor expression, promotes testosterone synthesis and secretion, and restores germ cell function, ultimately improving spermatogenesis in aluminum-exposed mice.

## Supporting information

S1 Raw Images Uncropped Western blot images. This file contains all original, uncropped Western blot scans related to the figures in the main text.
(PPTX)

S1 File Abstract Figure. NMN improves spermatogenesis in aluminum-exposed mice by suppressing inflammation and pyroptosis-related factors.
(TIF)
