# Supplementary material for: Nicotinamide mononucleotide improves spermatogenesis in aluminium-exposed mice by inhibiting NLRP3-mediated pyroptosis
Source: PLoS One. 2026 Jan 22;21(1):e0339020. doi: 10.1371/journal.pone.0339020 (PMC12826483; doi:10.1371/journal.pone.0339020)
Supplement: S1 Raw Images — This file contains all original, uncropped Western blot scans related to the figures in the main text. (PPTX) [file pone.0339020.s001.pptx]

## Slide 1
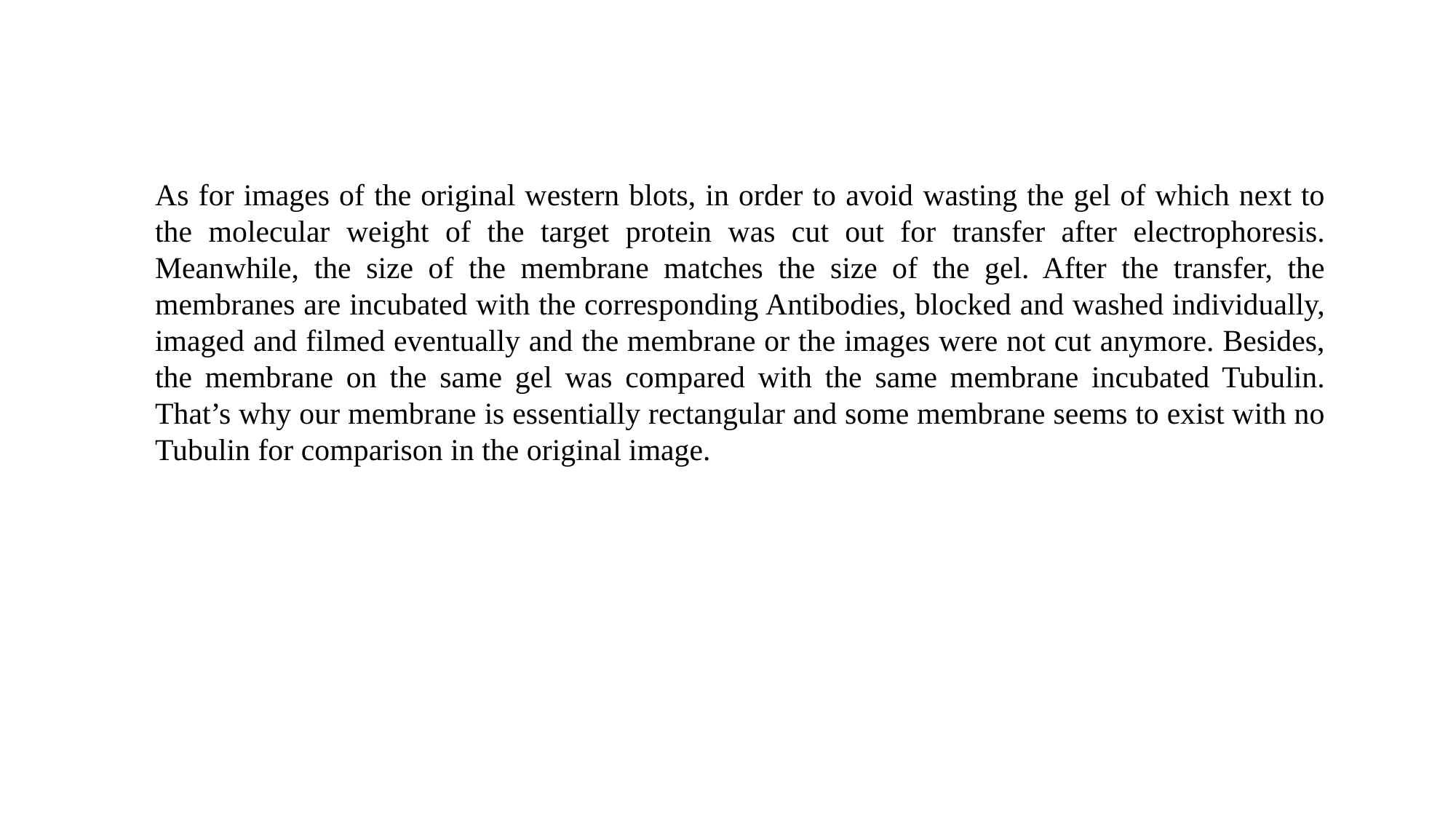

As for images of the original western blots, in order to avoid wasting the gel of which next to the molecular weight of the target protein was cut out for transfer after electrophoresis. Meanwhile, the size of the membrane matches the size of the gel. After the transfer, the membranes are incubated with the corresponding Antibodies, blocked and washed individually, imaged and filmed eventually and the membrane or the images were not cut anymore. Besides, the membrane on the same gel was compared with the same membrane incubated Tubulin. That’s why our membrane is essentially rectangular and some membrane seems to exist with no Tubulin for comparison in the original image.

## Slide 2
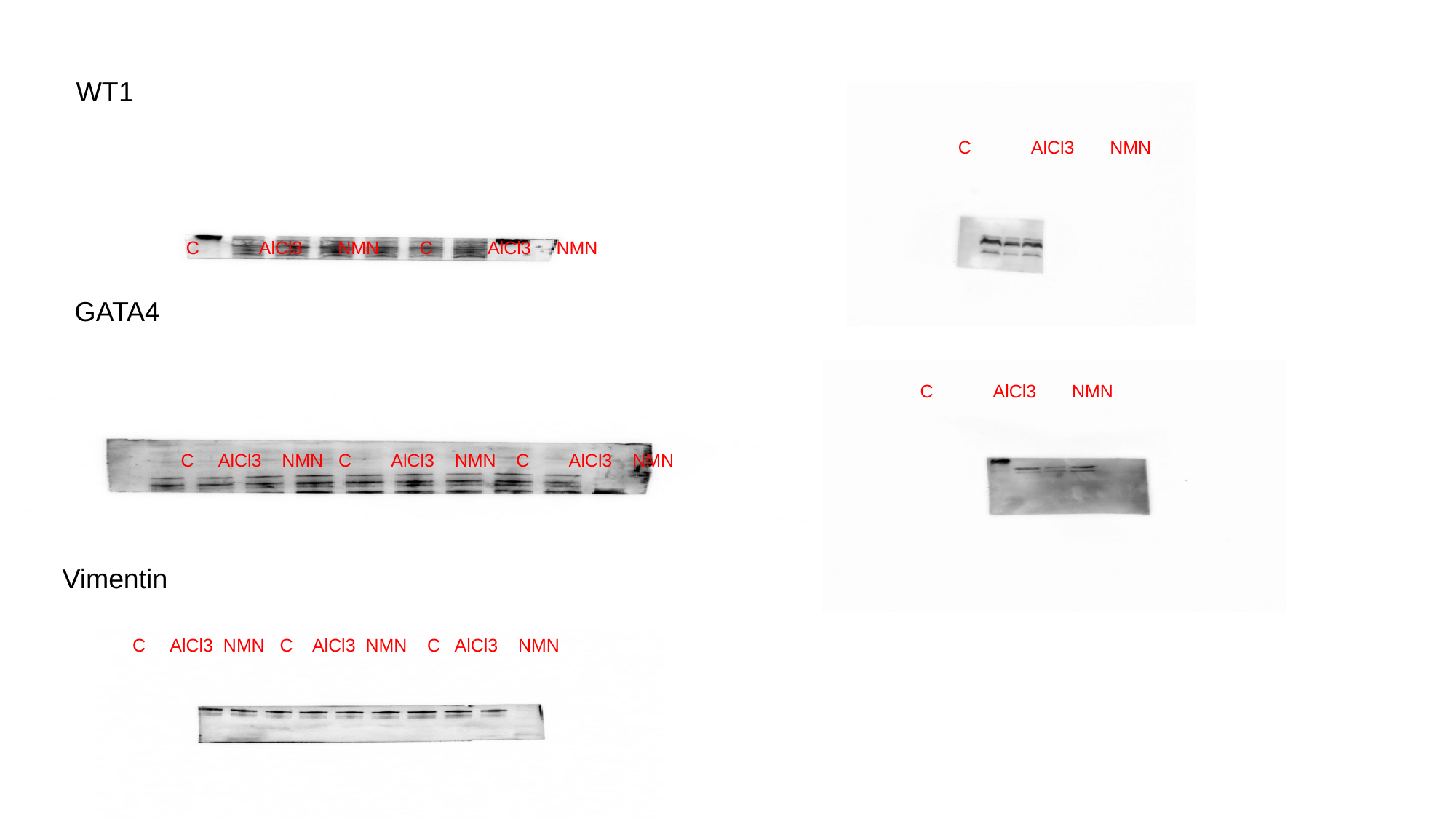

WT1
C AlCl3 NMN
C AlCl3 NMN C AlCl3 NMN
GATA4
C AlCl3 NMN C AlCl3 NMN C AlCl3 NMN
C AlCl3 NMN
Vimentin
C AlCl3 NMN C AlCl3 NMN C AlCl3 NMN

## Slide 3
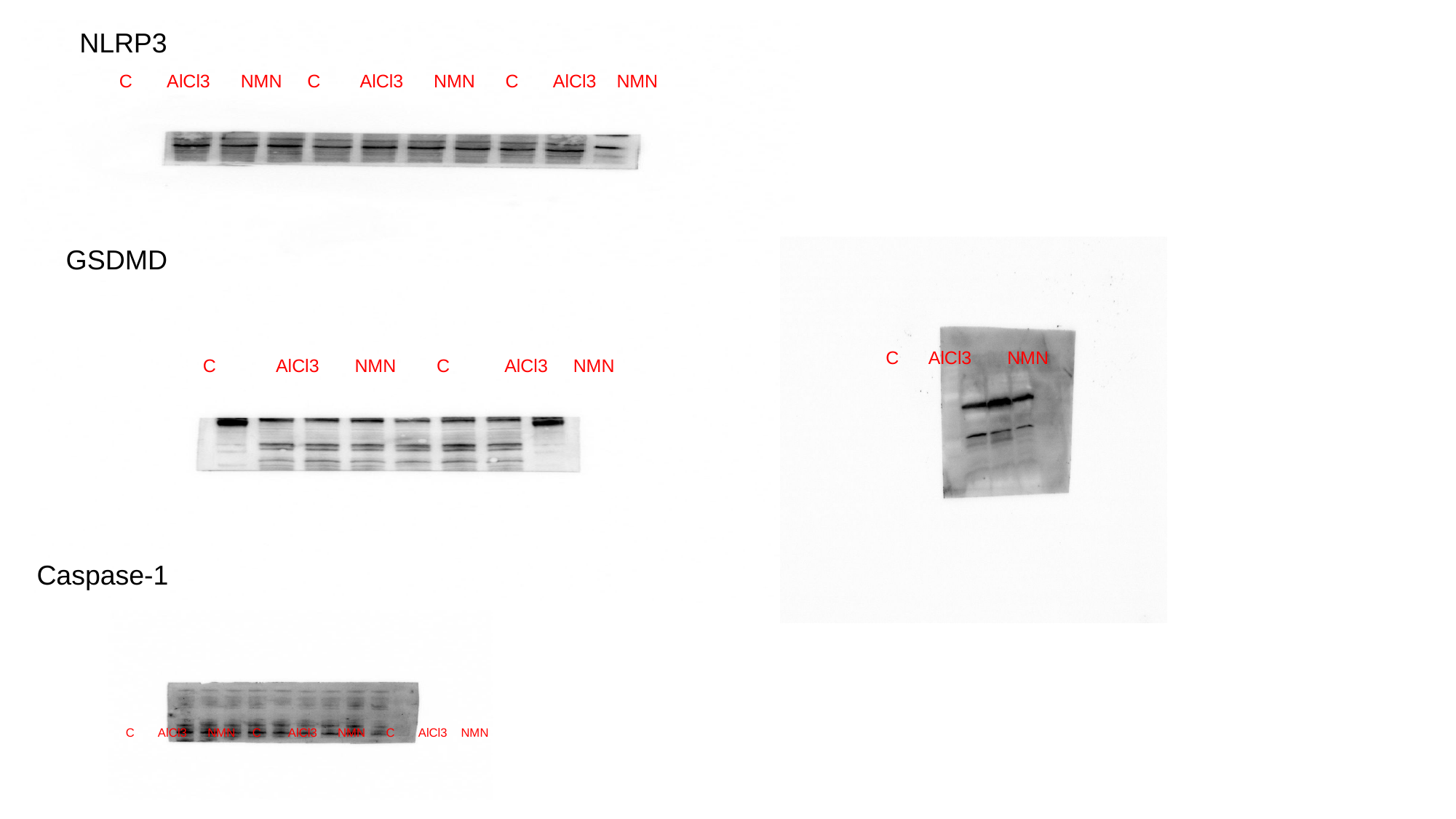

NLRP3
C AlCl3 NMN C AlCl3 NMN C AlCl3 NMN
GSDMD
C AlCl3 NMN
C AlCl3 NMN C AlCl3 NMN
Caspase-1
C AlCl3 NMN C AlCl3 NMN C AlCl3 NMN

## Slide 4
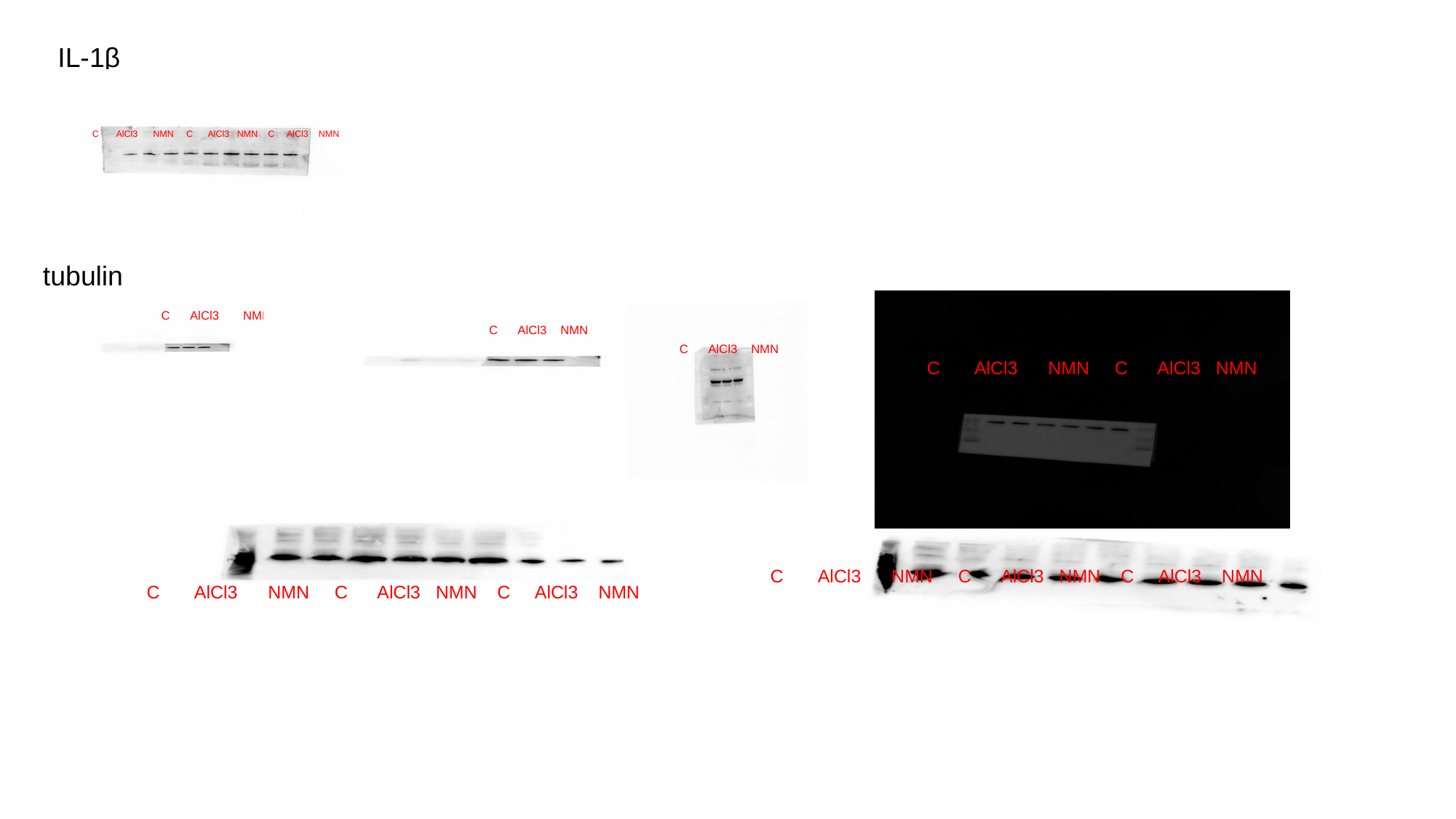

IL-1β
C AlCl3 NMN C AlCl3 NMN C AlCl3 NMN
tubulin
C AlCl3 NMN
C AlCl3 NMN
C AlCl3 NMN
C AlCl3 NMN C AlCl3 NMN
C AlCl3 NMN C AlCl3 NMN C AlCl3 NMN
C AlCl3 NMN C AlCl3 NMN C AlCl3 NMN
