# Supplementary figures and images for: Nicotinamide mononucleotide improves spermatogenesis in aluminium-exposed mice by inhibiting NLRP3-mediated pyroptosis
Source: PLoS One. 2026 Jan 22;21(1):e0339020. doi: 10.1371/journal.pone.0339020 (PMC12826483; doi:10.1371/journal.pone.0339020)

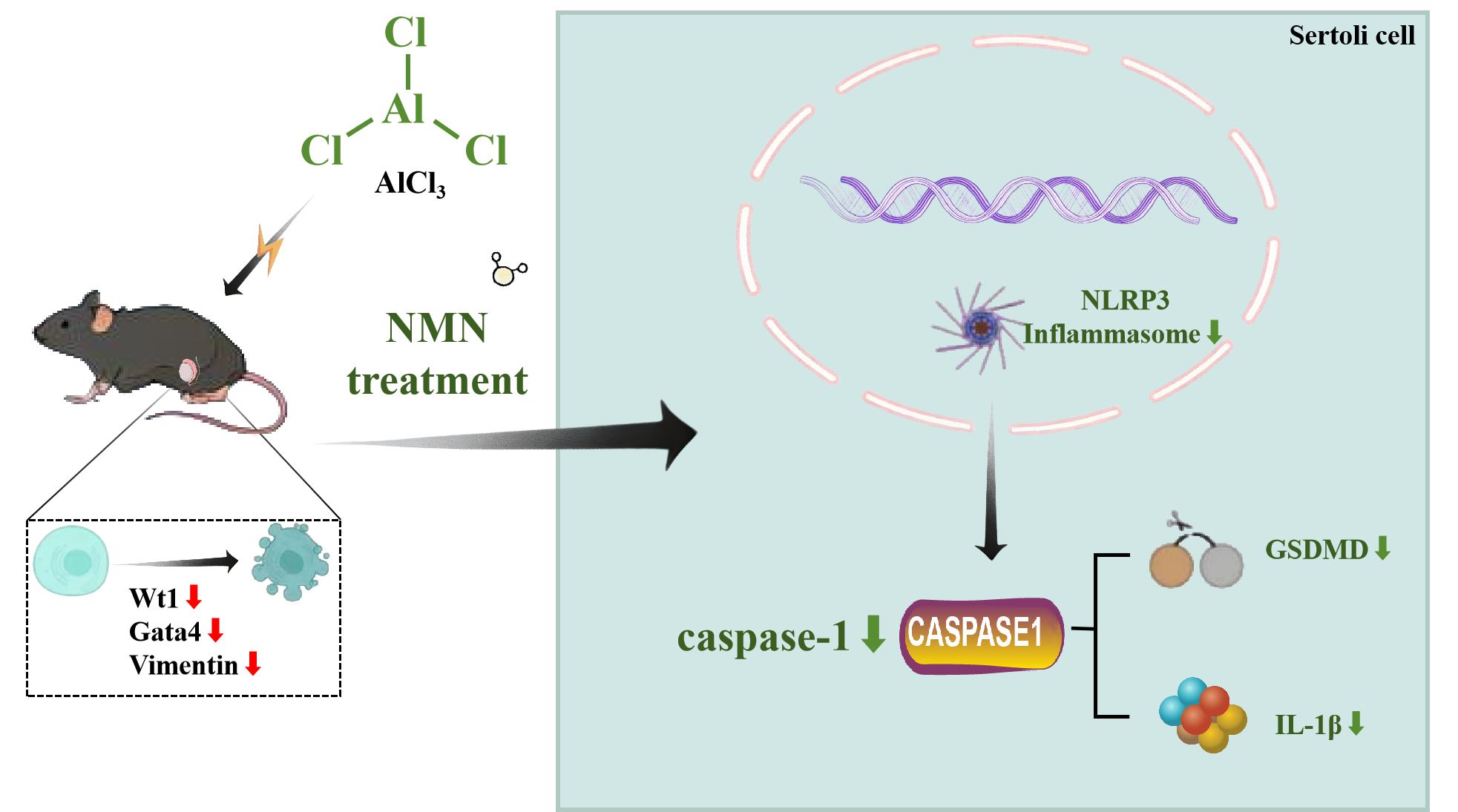

Supplement: S1 File — NMN improves spermatogenesis in aluminum-exposed mice by suppressing inflammation and pyroptosis-related factors. (TIF) [file pone.0339020.s002.tif]
